# Isothermal Vulcanization and Non-Isothermal Degradation Kinetics of XNBR/Epoxy/XNBR-g-Halloysite Nanotubes (HNT) Nanocomposites

**DOI:** 10.3390/ma14112872

**Published:** 2021-05-27

**Authors:** Seyed Mohamad Reza Paran, Ghasem Naderi, Elnaz Movahedifar, Maryam Jouyandeh, Krzysztof Formela, Xavier Colom, Javier Cañavate, Mohammad Reza Saeb

**Affiliations:** 1 Department of Polymer Processing, Iran Polymer and Petrochemical Institute, Tehran P.O. Box 14965/115, Iran; g.naderi@ippi.ac.ir (G.N.); e.movahedifar@ippi.ac.ir (E.M.); 2 Center of Excellence in Electrochemistry, School of Chemistry, College of Science, University of Tehran, Tehran P.O. Code 14176/14411, Iran; maryam.jouyande@gmail.com (M.J.); mrsaeb2008@gmail.com (M.R.S.); 3 Department of Polymer Technology, Faculty of Chemistry, Gdańsk University of Technology, Gabriela Narutowicza 11/12, 80-233 Gdańsk, Poland; 4 Department of Chemical Engineering, Universitat Politècnica de Catalunya Barcelona Tech, Terrassa, 08222 Barcelona, Spain; xavier.colom@upc.edu (X.C.); francisco.javier.canavate@upc.edu (J.C.)

**Keywords:** XNBR, epoxy, halloysite nanotubes, vulcanization kinetics, degradation, grafting

## Abstract

The effect of several concentrations of carboxylated nitrile butadiene rubber (XNBR) functionalized halloysite nanotubes (XHNTs) on the vulcanization and degradation kinetics of XNBR/epoxy compounds were evaluated using experimental and theoretical methods. The isothermal vulcanization kinetics were studied at various temperatures by rheometry and differential scanning calorimetry (DSC). The results obtained indicated that the n^th^ order model could not accurately predict the curing performance. However, the autocatalytic approach can be used to estimate the vulcanization reaction mechanism of XNBR/epoxy/XHNTs nanocomposites. The kinetic parameters related to the degradation of XNBR/epoxy/XHNTs nanocomposites were also assessed using thermogravimetric analysis (TGA). TGA measurements suggested that the grafted nanotubes strongly enhanced the thermal stability of the nanocomposite.

## 1. Introduction

The vulcanization degree of rubber compounds has a great role in determining the physical and mechanical properties of the manufactured rubber products [1]. The vulcanization reactions of the rubber compounds are under research in order to obtain useful insights about the complex mechanisms and kinetics involved [2]. Moreover in rubber compounds, the incorporation of nanofillers can change the cure kinetics of the systems because of the changes in the chemical and physical interactions that they produce [3,4,5,6]. For instance, Wu et al. reported that the incorporation of even a very low amount of graphene (GE) to natural rubber (NR), considerably influences the vulcanization kinetics of the system. An increase in the vulcanization rate, and decreases in scorch time and optimal cure time were correspondingly observed [7]. Another case of considerable change in the kinetics of an elastomer when adding a nanofiller was studied by Choi et al. [8]. They researched the vulcanization kinetics of nitrile butadiene rubber (NBR) nanocomposites with different organoclay contents using rheometry and differential scanning calorimetry (DSC) indicating a remarkable decrease in the scorch time without any significant changes in the optimal cure time and torque values. DSC measurements also unveiled a lower activation energy in the NBR/organoclay nanocomposites compared to the pristine NBR. Lipińska et al. studied the cure kinetics of carboxylated nitrile butadiene rubber (XNBR) nanocomposites containing layered double hydroxides (Mg-Al-LDHs) and reported lower ranges of curing temperatures as the Mg:Al ratio increased [9]. However, the apparent activation energy decreased with the increase of the Mg:Al ratio.

XNBR is a special type of nitrile butadiene rubber (NBR) in which the main polymer backbone is modified with a carboxyl functional group [10]. Generally XNBR compounds have higher tear and abrasion resistance in comparison with the NBR compounds [11]. Literature surveys show that the XNBR compounds containing various nanofillers are useful materials to make rubber parts with higher physical and mechanical properties [11,12]. However, as mentioned above, the introduction of nanofillers into the XNBR matrix influences the vulcanization behavior of the rubber compound [13]. Chudzik et al. [14] studied the effect of modified epoxy resin on the cure state and adhesion properties of various rubber compounds such as XNBR. They reported that the introduction of modified epoxy resin into the XNBR leads to a higher adhesion between the rubber and silver wire. Since the vulcanization of the rubber compounds is a key factor influencing the properties of the material and the addition of nanofillers has an important effect on the kinetics and mechanisms of vulcanization, the interest on the detailed knowledge of these phenomena is paramount.

In previous research, we studied XNBR/epoxy nanocomposites containing different amounts of XNBR grafted halloysite nanotubes (XHNTs). Our findings indicated that the addition of XHNTs enhanced the mechanical and dynamic-mechanical properties of the XNBR/epoxy matrix [15]. The results of the cure rheometer analysis confirmed the enhanced cure characteristics and cross-link density of XNBR/epoxy/XHNT nanocomposites due to possible interactions between the surface modified nanotubes and the XNBR/epoxy matrix. Morphological observations of XNBR/epoxy nanocomposites containing various XHNTs show that the nanotubes have a uniform dispersion state in the polymer matrix which leads to higher mechanical properties.

After these previous studies, increasing the knowledge of the effect of various concentrations of XHNTs on the vulcanization and degradation of XNBR/epoxy nanocomposites was the main objective of the present research. In order to achieve that, XNBR/epoxy/XHNTs nanocomposites containing various loadings of nanotubes were prepared and the cure kinetics of these compounds was experimentally evaluated at various temperatures using an oscillating disc rheometer (ODR) and an isothermal differential scanning calorimeter. Moreover, the degradation behavior of the prepared nanocomposites was monitored by thermogravimetric analysis (TGA) at various heating rates. Various theoretical approaches were applied to the obtained experimental data to evaluate the vulcanization and degradation kinetics parameters.

## 2. Theoretical Background

### 2.1. Vulcanization Kinetics

The vulcanization reaction of a rubber compound can be evaluated through using a differential equation on the basis of time and temperatures of the reaction as the following equation [16,17,18]:

(1) $$\frac{d\alpha }{\mathrm{dt}}=K\left(T\right)f\left(\alpha \right)$$

where α is the degree of cure, dα/dt is the curing rate, t is time, K is the kinetic constant at temperature T and f(α) is function related to the adopted model. The degree of cure parameter is defined as the following relation in the oscillating disc rheometer (ODR) analysis [19]:

(2) $$\alpha =\frac{M_{t}-M_{0}}{M_{\infty }-M_{0}}$$

where M_t_ is the torque value at time t, M_0_ and M_∞_ are the torque values at time zero and the end of vulcanization reaction, respectively. However, the degree of cure can be calculated from the DSC measurements as the following equation [20,21]:

(3) $$\alpha =\frac{\Delta H_{t}}{\Delta H_{\infty }}$$

where ∆H_t_ and ∆H_∞_ are the heat of reaction at time *t* and total heat of reaction, respectively.

The kinetic constant, K(T), is determined by the Arrhenius equation [22,23]:

(4) $$K\left(T\right)=A\exp\left(-\frac{E_{\alpha }}{\mathrm{RT}}\right)$$

where R is the universal gas constant, A and E_α_ are the kinetic parameters, the pre exponential factor and activation energy, respectively [24]. The overall vulcanization reaction rate can represented as [25]:

(5) $$\frac{d\alpha }{\mathrm{dt}}=A\exp\left(-\frac{E_{\alpha }}{\mathrm{RT}}\right)f\left(\alpha \right)$$

where f(α) is the vulcanization reaction model. A simple integration of above equation by considering the independency of A and E on the vulcanization temperature, yields the relation between time and heating temperature of the curing process [26]:

(6) $$\ln t_{\alpha ,i}=\ln\left[\frac{g\left(\alpha \right)}{A_{a}}\right]+\frac{E_{\alpha }}{RT_{i}}$$

where g(α) is the integral form of the reaction model, tα, i is the time needed to reach the conversion to a specific value at temperature Ti. It is obvious from Equation (6) that the plots of lntα,i vs. 1/T_i_ are lines with the slope of E_α_/R.

Vulcanization reaction of rubber compounds can be represented by n^th^ order model as the following equation [27,28]:

(7) $$\frac{d\alpha }{\mathrm{dt}}=K\left(T\right){\left(1-\alpha \right)}^{n}$$

The logarithmic form of the above equation can be shown as:

(8) $$\ln\left(\frac{d\alpha }{\mathrm{dt}}\right)=\ln\left(K\right)+\mathrm{nln}\left(1-\alpha \right)$$

The resulting graph of ln(dα/dt) vs. ln(1-α) is a line the slope of which gives the value of n and the intercept of ln(K). Whereas, the activation energy can be calculated from the slope of the following linear equation, which represents the relation between ln(K) and 1/T:

(9) $$\ln\left(K\right)=\ln\left(A\right)-\frac{E_{\alpha }}{\mathrm{RT}}$$

The molecular approach about the vulcanization process of a rubber compound proposed a mix of reacted and unreacted reactive sites in a curing system which controls the vulcanization reaction [29]. The single step autocatalytic reaction model can be applied to determine the parameters of the vulcanization reaction [30,31]:

(10) $$\frac{d\alpha }{\mathrm{dt}}=K\left(T\right)\alpha^{m}{\left(1-\alpha \right)}^{n}$$

where α^m^ and (1-α)^n^ represented the reacted and unreacted sites in the vulcanization reaction, respectively. It should be noted that the reaction order m lies between 0 to 1 and n ≥ 1 [32,33]. The K(T) is constant for isothermal conditions which easily allows the values of m and n to be determined.

### 2.2. Degradation Kinetics

The rate of thermal decomposition in a polymer system could be represented as Equation (1). However, there is a difference which the parameter, α, is defined as the extent of polymer degradation (partial mass loss) as the following equation [34,35]:

(11) $$\alpha =\frac{W_{0}-W_{t}}{W_{0}-W_{f}}$$

where W_0_, W_t_ and W_f_ are initial, time and final weights of the polymer, respectively [36]. Activation energy for a given decomposition information based on thermogravimetric analysis (TGA) could be calculated by a differential isoconversional technique such as the Friedman method which is represented as the following relation [37,38]:

(12) $$\ln\left(\frac{d\alpha }{\mathrm{dt}}\right)=\ln\left[f\left(\alpha \right)A_{\alpha }\right]-\frac{E_{\alpha }}{{\mathrm{RT}}_{\alpha }}$$

where the activation energy of thermal decomposition reaction can be determined from the slope of ln(dα/dt) against 1/Tα at a certain partial mass loss. As the numerical differentiation of TGA data cause some deviations and inaccuracies to predict the degradation kinetics, the integral isoconversional methods were preferred by Kissinger–Akahira–Sunose (KAS) [39]:

(13) $$\ln\left(\frac{\beta_{i}}{T_{\alpha ,i}^{2}}\right)=\mathrm{Const}-\left(\frac{E_{\alpha }}{{\mathrm{RT}}_{\alpha }}\right)$$

where βi = dT/dt, is defined as heating rate and activation energy evaluated from the slope of the resulting line of $\ln\left(\frac{\beta_{i}}{T_{\alpha ,i}^{1.92}}\right)$ vs. 1/T.

Ozawa–Flynn–Wall proposed an integral based method which Eα calculated from the slope of ln(βi) with respect to 1/Tα at any certain partial mass loss as the following equation [40]:

(14) $$\ln\left(\beta_{i}\right)=\mathrm{Const}-1.052\left(\frac{E_{\alpha }}{{\mathrm{RT}}_{\alpha }}\right)$$

## 3. Materials and Methods

Carboxylated nitrile butadiene rubber (XNBR), Krynac X160, was purchased from Lanxess Elastomers (Leverkusen, Germany). The XNBR contains 32.5% by weight of acrylonitrile and 1% by weight of the carboxylic acid group.

The epoxy resin was KER828, a diglycidyl ether of bisphenol A (DGEBA) type, with an epoxy group content of 5260–5420 mmol/kg. The epoxy resin was supplied by Kumho P&B chemicals (Seoul, South Korea).

XNBR-grafted halloysite nanotubes (XHNTs) were synthesized in accordance with our previous research works using halloysite nanotubes (HNTs). The ultrafine grade HNTs were obtained from Imerys Tableware Asia Limited (Auckland, New Zealand) [15].

Other ingredients such as zinc oxide and stearic acid were laboratory reagent grades from Merck Co. (Frankfurt, Germany) and used as received.

Various formulations of XNBR/epoxy/XHNT nanocomposites in accordance with Table 1 were prepared on a laboratory open two roll mill mixer, running at rotor speed ratio of 1:1.2 for 10 min at 40 °C. For this purpose, the XNBR was first masticated for 1 min and then the epoxy resin was added to the rubber with a proportion of 85/15 of rubber to resin. The XHNTs were introduced into the rubber mixture after 2 min of mixing process and the mixing was continued for 5 min. The ZnO and acid stearic were incorporated into the nanocomposite and mixed for 3 min at the final stage. The ZnO can react with the carboxylic group of XNBR and acts as a curing agent for this rubber.

The cure behavior of prepared nanocomposites were studied by using a Monsanto Oscillating Disc Rheometer R-100 (ODR, MonTech, Columbia City, IN, USA) operated at different temperatures 170, 180, 190 and 200 °C with 3° arc at a period of 30 min in accordance with ASTM D2084.

The vulcanization characteristics of the prepared samples was also determined from isothermal differential scanning calorimeter (DSC) using a Netzsch-Maia-200F3 (NETZSCH Premier Technologies, Exton, PA, USA) under a nitrogen atmosphere operated at various temperatures 170, 180, 190 and 200 °C at a period of 30 min.

Thermogravimetric analysis (TGA, NETZSCH Premier Technologies, Exton, PA, USA) for various XNBR/epoxy/XHNT nanocomposites was conducted by Netzsch STA instruments, NETZSCH Premier Technologies, Exton, PA, USA, 409 PC thermogravimetric analyzer under nitrogen atmosphere provided by fixed gas flow rate of 100 cm^3^/min and a temperature range of 30–600 °C. The heating rates were adjusted at 5, 10, 15 and 20 °C/min.

## 4. Results and Discussion

### 4.1. Cure Characteristics

**Figure 1:**
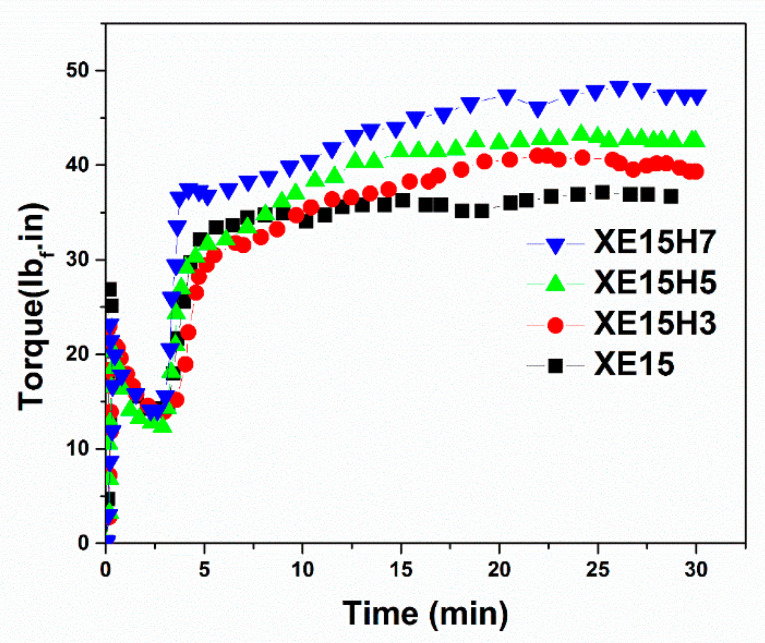
Cure behavior of XNBR/epoxy nanocomposites containing various XHNT loadings.

The vulcanization behavior of XNBR/epoxy nanocomposites containing different concentrations of XHNTs at temperature of 170 °C is depicted in Figure 1. We can see that the higher concentrations of XHNTs cause higher torque values at the initial and final steps of curing of nanocomposites. The parameters related to the vulcanization of XNBR/epoxy compounds and its nanocomposites at various heating temperatures are displayed in Table 2. The results show that the scorch time and optimal cure time reduced with the introduction of XNHTs into the XNBR/Epoxy matrix. It may have contributed to the interactions between the XHNTs and XNBR/epoxy matrix which leads to a higher values in the torque rheometer and curing rate [41]. It is assumed that some possible interactions between the nanotubes and polymer matrix create immobility in the polymer chains which leads to an accelerated cure reaction.

### 4.2. Vulcanization Kinetics

**Figure 2:**
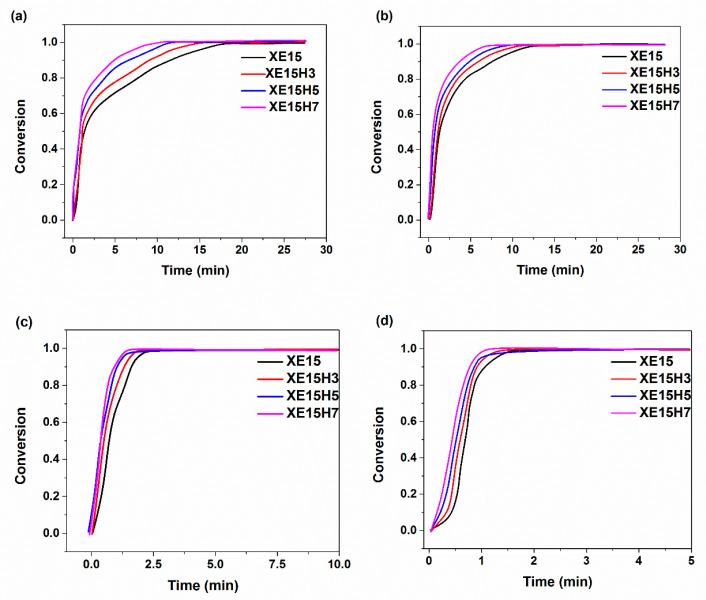
Variation the degree of cure parameter with respect to the time for various XNBR/epoxy/XHNTs at different heating temperatures (**a**) 170 °C (**b**) 180 °C (**c**) 190 °C (**d**) 200 °C calculated by using Equation (2).

The variation of the degree of curing parameter vs. time calculated from rheometer analysis at various heating temperatures was investigated in Figure 2 for various XNBR/epoxy/XHNT nanocomposites. The results indicated that for a given heating temperature, the degree of curing rapidly increased after the activation of the curing reaction. After the initial step, the degree of curing slowly increased until reaching a constant value at the final stage. A shift in the degree of cure parameter to higher values at the initial stage of vulcanization reaction was attributed to the chain extension and cross-linking of XNBR chains [42]. However, the cross-linking reactions retarded the movement of reacting molecules which resulted in a decrease in the rate of conversion [43].

The effect of incorporation of XHNTs into the XNBR/epoxy matrix can be monitored in Figure 2a–d. A higher content of nanotubes increased the cure rate and degree of conversion at the initial stage of the vulcanization reaction.

**Figure 3:**
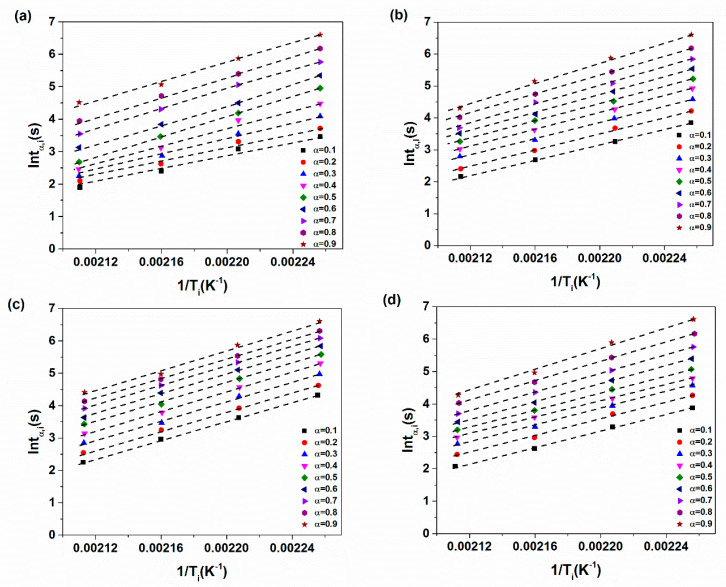
Plots of lntα,i vs. 1/Ti for various XNBR/epoxy/XHNTs nanocomposites (**a**) XE15 (**b**) XE15H3 (**c**) XE15H5 (**d**) XE15H7.

**Figure 4:**
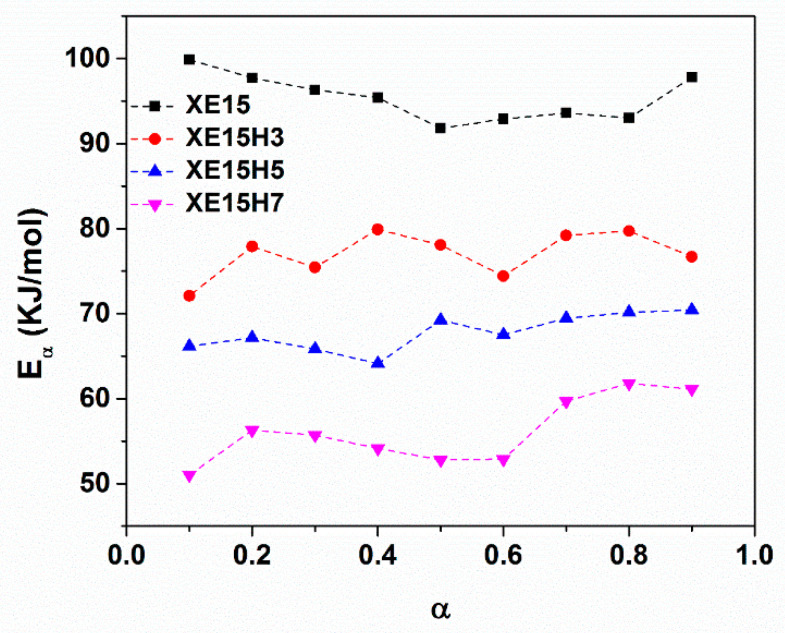
Variation of activation energy at different cure conversion for various XNBR/epoxy/XHNT nanocomposites.

Figure 3 shows the plots of lntα,i as a function heating temperature (1/Ti) for various prepared nanocomposites. We can see that for all samples the plots of lntα,i vs. 1/Ti is a line for every specified conversion the slope of which represented the activation energy. The resulted activation energy as a function of cure conversion for all prepared samples is depicted in Figure 4. The results indicated that the activation energy decreased with higher concentrations of XHNTs which is attributed to the effect of XNBR-grafted nanotubes on the vulcanization reaction of nanocomposites [44]. Therefore, lower energies are needed to complete the vulcanization process.

**Figure 5:**
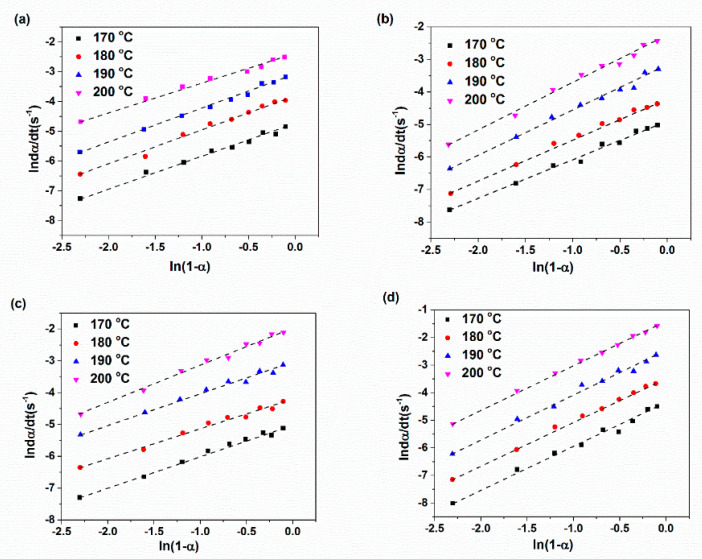
Plots of ln(dα/dt) vs. ln(1-α) for various XNBR/epoxy/XHNT nanocomposites based on n^th^ order model for: (**a**) XE15; (**b**) XE15H3; (**c**) XE15H5; (**d**) XE15H7.

**Figure 6:**
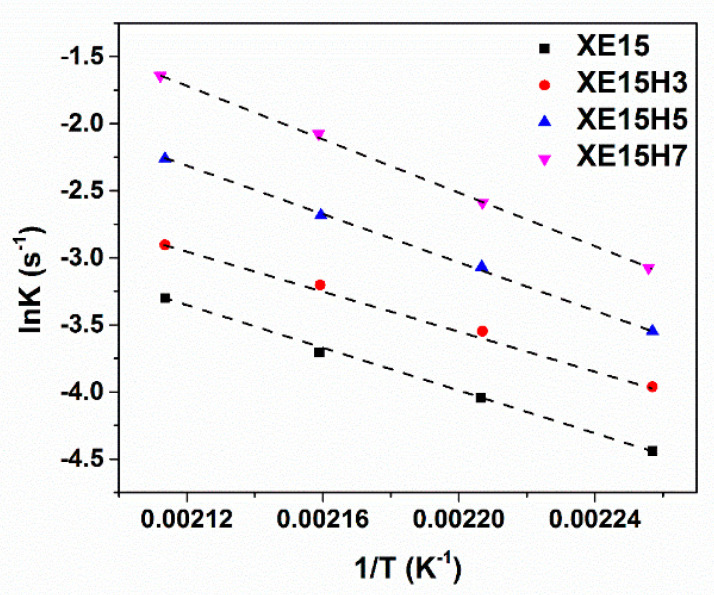
Plots of ln(K) with respect to 1/T for various prepared nanocomposites.

**Figure 7:**
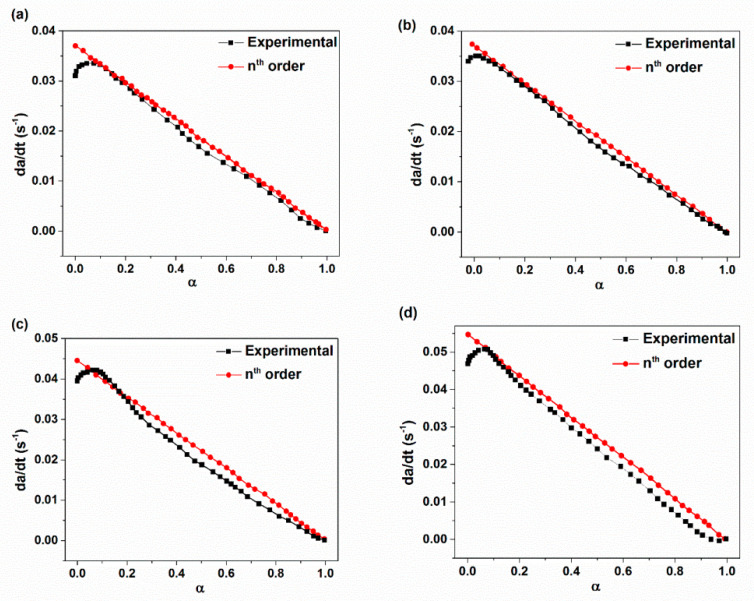
Comparison of experimental conversion rate with predicted ones through using n^th^ order model with respect to cure conversion parameter for: (**a**) XE15; (**b**) XE15H3; (**c**) XE15H5; (**d**) XE15H7.

The plots of ln(dα/dt) with respect to ln(1-α) and ln(K) versus 1/T are demonstrated in Figure 5 and Figure 6 for various XNBR/epoxy/XHNT nanocomposites. The calculated kinetic parameters based on n^th^ order model for various prepared nanocomposites are summarized in Table 3. The results obtained indicated higher values of rate constant with higher temperatures in which this effect is more pronounced with the incorporation of XHNTs into the XNBR/epoxy matrix. Furthermore, the reaction order, n, lies between 1 and 2 for all samples and increased with temperature. It should be noted that the reaction order increases with the introduction of XHNTs into the rubber matrix which is attributed to the effect of XHNTs on the vulcanization reaction [45]. As can be observed in Figure 7, there is a difference between the predicted conversion rate (dα/dt) curves from the n^th^ order kinetic model and experimental values at most regions of the conversion parameter.

The results of vulcanization analysis for various XNBR/epoxy/XHNTs through using the autocatalytic approach are presented in Table 4. It should be noted that the kinetic parameters were evaluated using a non-linear regression analysis. As can be seen, the rate constant increased with the higher heating temperature. However, the values of reaction order, m and n, varies with temperature and XHNT loading. One can see that the reaction order, n, lies between 1 and 2 which increases with temperature and XHNT loading whereas the reaction order, m, lies between 0 and 1 which shows a more variations with respect to the XHNTs content compared to the n parameter. As indicated in Table 4, the activation energy required for vulcanization reaction decreased with higher concentration of XHNTs such as the results of n^th^ order model.

**Figure 8:**
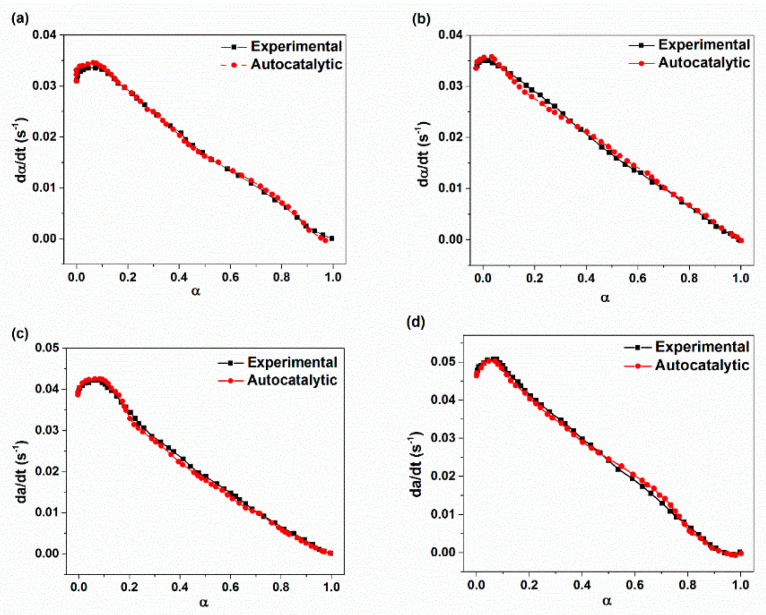
Comparison of experimental conversion rate with predicted ones through using autocatalytic approach with respect to cure conversion parameter for: (**a**) XE15; (**b**) XE15H3; (**c**) XE15H5; (**d**) XE15H7.

Figure 8 compares the plot of the conversion rate (dα/dt) resulting from experimental and theoretical values based on the autocatalytic approach. It is evident from Figure 8 that there is a good agreement between the experimental values and predicted conversion rate for prepared nanocomposites. However, there are some deviations from the experimental conversion rate with higher XHNT loading especially at the initial state of vulcanization reaction. This may be due to the effect of XHNTs on the reaction process resulting from some polymer–filler interactions [15,46,47].

As previously discussed, and as shown in Table 3 and Table 4 for the other proposed models, the Eα decreases as the XHNT content increases. This means that the vulcanization process is much faster with XHNT content because of XNBR grafted nanotubes on the vulcanization reaction of nanocomposites. The nanotubes act as an accelerator for the whole reaction of vulcanization according to all the results obtained.

### 4.3. Vulcanization Kinetics by Differential Scanning Calorimetry (DSC)

**Figure 9:**
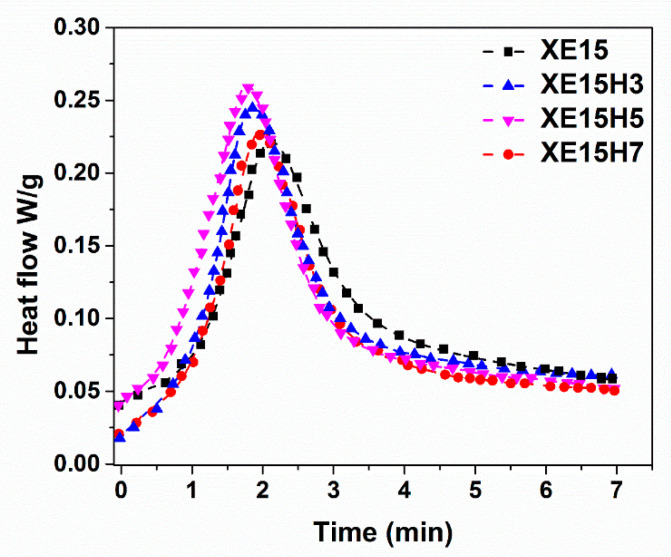
Typical isothermal differential scanning calorimetry (DSC) thermograms of various XNBR/epoxy/XHNT nanocomposites at 170 °C.

As the DSC measurement is a useful technique to determine the reaction kinetics parameters, it is used in the form of isothermal method to display the vulcanization kinetics of XNBR/epoxy/XHNT nanocomposites. Figure 9 represents a typical isothermal DSC scan for various XNBR/epoxy/XHNTs nanocomposites at 170 °C. The rate and degree of vulcanization reaction can be predicted from the exothermic peak position and the area under the DSC curves [48,49]. A comparison among DSC curves of various prepared nanocomposites suggested that the nanocomposites containing a higher loading of nanotubes exhibits a higher cross-linking and more rapid vulcanization process [50,51].

**Figure 10:**
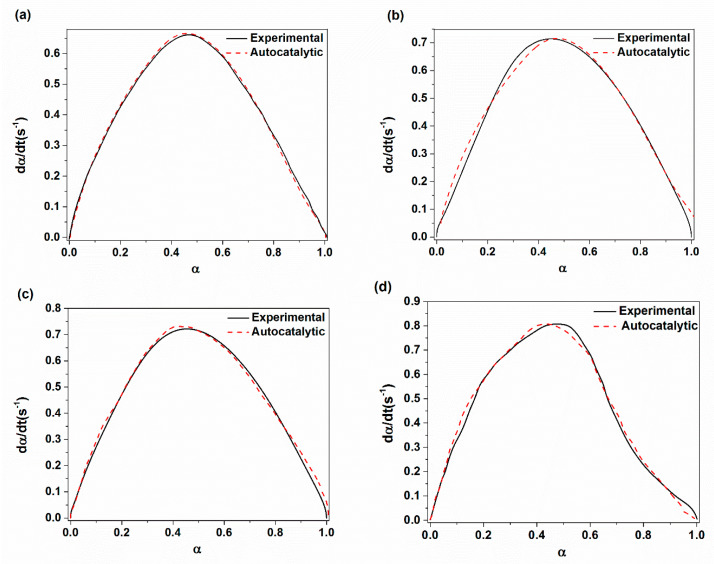
Comparison of experimental conversion rate with predicted ones through using autocatalytic ap-proach and isothermal DSC curves with respect to cure conversion parameter for: (**a**) XE15; (**b**) XE15H3; (**c**) XE15H5; (**d**) XE15H7.

To study of kinetics of vulcanization of various prepared nanocomposites, the total enthalpy of curing and fraction of heat released at time, t, were calculated from isothermal DSC curves. Then the degree of vulcanization and rate of reaction were calculated to determine the kinetics parameters in accordance with the autocatalytic approach. The kinetics parameters were evaluated through using non-linear regression analysis which is presented in Table 5. The results indicated that the kinetics parameters obtained from isothermal DSC analysis show a direct proportionality with temperature such as rheometer analysis. However, there is a difference between the resulting cure kinetics parameters from DSC curves and rheometer analysis due to the different nature of theoretical background of two methods of analysis [8,52]. The predicted cure rate for various XNBR/epoxy/XHNT nanocomposites obtained from using the autocatalytic approach and experimental values are depicted in Figure 10. It is clear that there is a good agreement between the predicted and theoretical values, indicating the suitability of the autocatalytic approach to study the vulcanization process of prepared nanocomposites.

### 4.4. Thermal Degradation Kinetics

**Figure 11:**
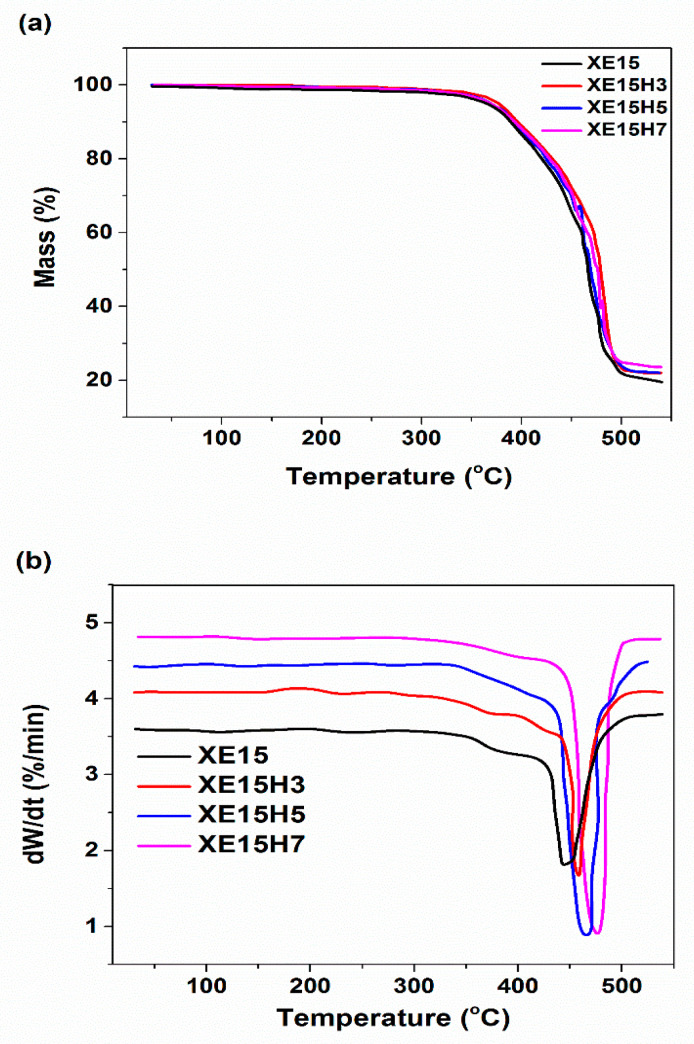
(**a**) Thermogravimetric analysis (TGA) and (**b**) DTG thermograms of various XNBR/Epoxy/XHNTs at heating temperature of 5 °C/min.

The results of thermogravimetric analysis (TGA) for various XNBR/epoxy/XHNTs conducted at heating rate of 5 °C/min were evaluated in Figure 11. The resulted degradation parameters of various prepared nanocomposites were presented in Table 6. It is observed that the major decomposition of various XNBR/epoxy/XHNT nanocomposites occurs at temperatures higher than 350 °C.

**Figure 12:**
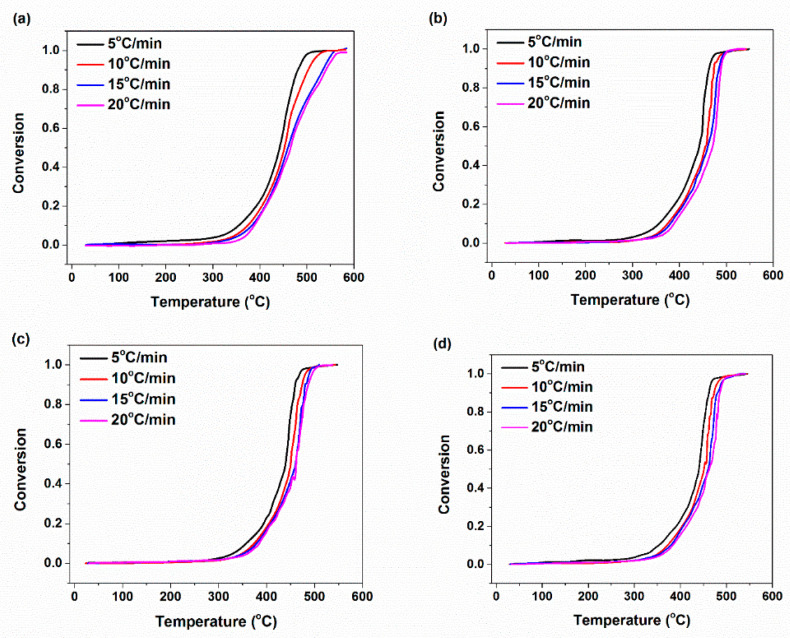
Degradation conversion plots of XNBR/epoxy/XHNT nanocomposites at various heating rates (**a**) XE15 (**b**) XE15H3 (**c**) XE15H5 (**d**) XE15H7.

The effects of heating rate and XHNTs loading on the partial mass loss parameter, α, are depicted in Figure 12 for various prepared nanocomposites. It is evident that the shape of the resulting graphs is similar for all prepared samples which indicated that the XHNTs does not affect the degradation mechanism of XNBR/epoxy matrix [53]. However, the results indicated that the higher heating rate leads to a higher temperature to complete degradation of the prepared samples.

**Figure 13:**
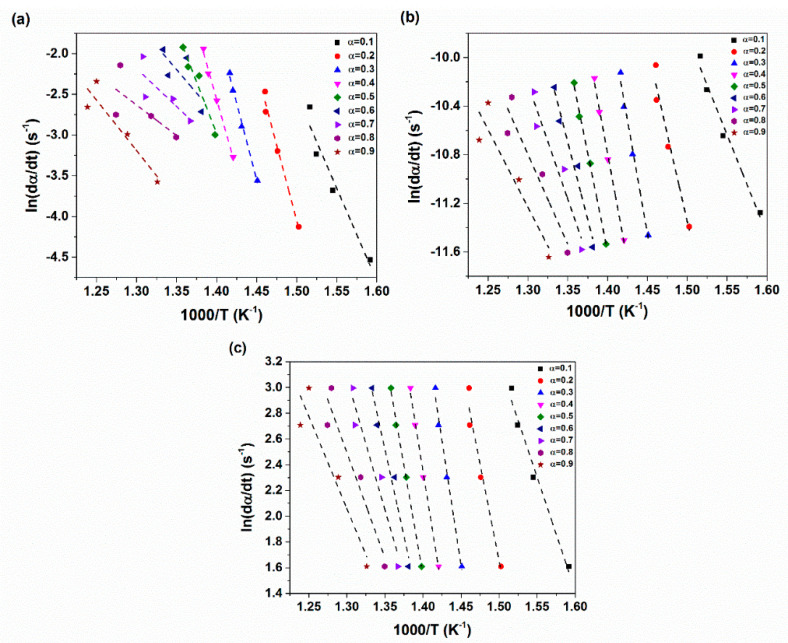
Typical isoconversional graphs for XNBR/epoxy/XHNT nanocomposites through using (**a**) Friedman (**b**) Kissinger–Akahira–Sunose (KAS) (**c**) Ozawa-Flynn–Wall (OFW) methods.

In order to calculate the activation energy required for degradation reactions, the fitted straight lines at various partial mass loss are presented in Figure 13 on the basis of the Friedman, Kissinger–Akahira–Sunose (KAS) and Ozawa-Flynn–Wall (OFW) models. It is clear from Figure 13a that the fitted straight lines through using the Friedman method has some errors which makes this model unable to precisely predict the degradation mechanism of prepared nanocomposites. However, the resulting straight lines by using the KAS and OFW models in Figure 13b,c are parallel together at most partial mass loss values and well fitted to the experimental data which are attributed to the integral base of these models [54].

**Figure 14:**
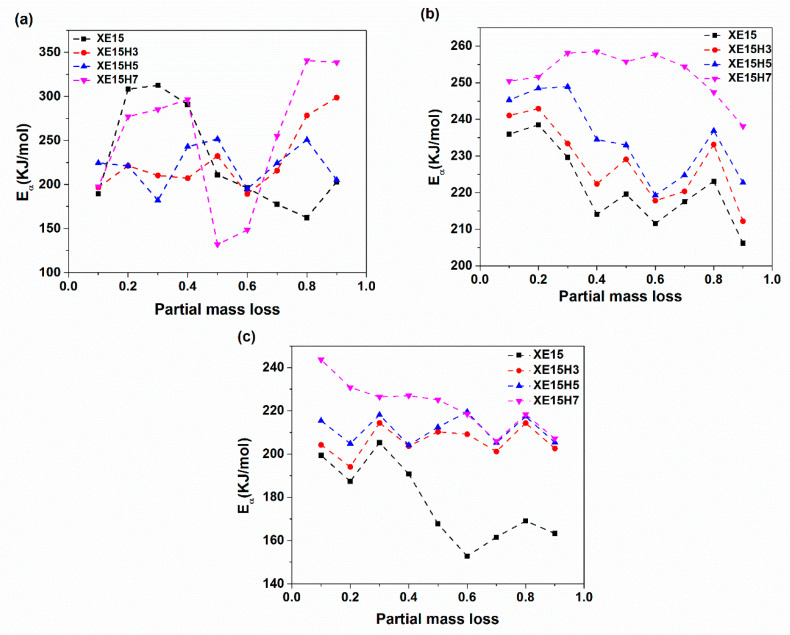
Variation of activation energy with the partial mass loss parameter and XHNT concentration calculated through using (**a**) Friedman (**b**) KAS and (**c**) OFW methods.

Figure 14 demonstrates the variation of activation energy for degradation of XNBR/Epoxy/XHNTs nanocomposites with partial mass loss parameter. The results indicated that the activation energy calculated through using the Friedman method show disordered variations with the partial mass loss. However, the graph of activation energies derived from the KAS and OFW methods have more regularity at various partial mass losses. As can be seen in Figure 14, the nanocomposites containing higher concentration of XHNTs show higher activation energies at a whole range of partial mass loss. In other word, the nanotubes act as a retarder for the degradation reaction of XNBR/epoxy compound which can be attributed to its physical structure and probable interactions with the polymer matrix [55].

The calculated degradation kinetic parameters based on autocatalytic approach are presented in Table 7. We can see that the frequency factor, A, which is directly dependent on the activation energy [56], shows the same trend and increases with the incorporation of XHNTs into the XNBR/epoxy matrix. The results of reaction orders, m and n, indicated that this parameters decrease with the XNTs loading which is attributed to the retardation effect of nanotubes in the degradation mechanism of the polymer matrix [57].

**Figure 15:**
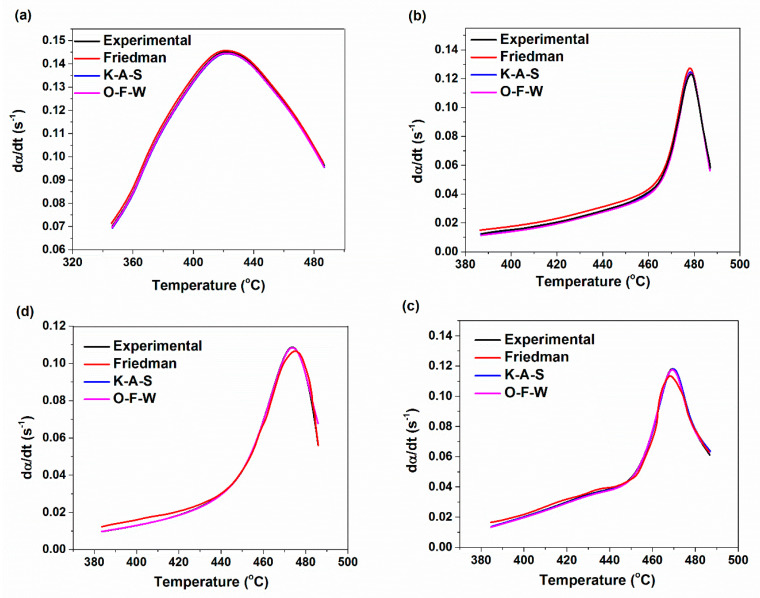
Predicted kinetics curves based on the autocatalytic degradation reaction model for XNBR/epoxy/XHNT nanocomposites containing various nanotube loadings at the heating rate of 5 °C/min for: (**a**) XE15; (**b**) XE15H3; (**c**) XE15H5 and (**d**) XE15H7.

The predicted rate of degradation reaction by using theoretical models and experimental data are compared in Figure 15. The results indicated that there is a good agreement between the theoretical and experimental values of the rate of degradation reaction for various XNBR/epoxy/XHNTs through using the KAS and OFW models. However, there are some deviations from experimental values by Friedman model especially with higher XHNTs loading at the early stage of the degradation mechanism.

## 5. Conclusions

XNBR/epoxy nanocomposites containing different concentrations of XNBR-grafted halloysite nanotubes (XHNTs) were prepared by using laboratory two roll mills.

The cure rheometer investigations at various temperatures demonstrated that the introduction of XHNTs into the XNBR/epoxy matrix causes a rise in the maximum torque values while it decreases the scorch and optimal cure times. The vulcanization kinetics study revealed that the cure reaction of prepared XNBR/epoxy/XHNTs nanocomposites follows the autocatalytic model and the n^th^ order model could not precisely predict the vulcanization mechanism. However, there are some differences between the resulting vulcanization reaction parameters calculated through using a cure rheometer and isothermal DSC analysis. The results suggested that the incorporation of XHNTs into the polymer matrix cause a change in the vulcanization reaction which leads to a decrease in the activation energy parameter. From these results, the conclusion is that the addition of XHNTs accelerates the vulcanization process.

The study of the degradation mechanism of prepared nanocomposites using non-isothermal TGA measurements indicated that the nanotubes act as a retarder in the degradation reaction with a higher activation energy. The results suggested that the incorporation of XNBR-grafted halloysite nanotubes into the XNBR/epoxy compound will produce a nanocomposite with better vulcanization behavior and higher thermal stability.

## Figures and Tables

**Table 1:** Formulations of carboxylated nitrile butadiene rubber (XNBR)/epoxy nanocomposites containing various functionalized halloysite nanotube (XHNT) loadings.

Item	Designation	XNBR/Epoxy (phr)	HNT (phr)	ZnO (phr)	Stearic Acid (phr)
1	XE15	100	0	6	2
2	XE15H3	100	3	6	2
3	XE15H5	100	5	6	2
4	XE15H7	100	7	6	2

**Table 2:** Cure parameters of XNBR/epoxy/XHNT nanocomposites at various heating rates.

Designation	Heating Temperature (°C)	*M_H_* (N.m)	*M_L_* (N.m)	(*M_H_* − *M_L_*) (N.m)	$t_{s^{2}}$ (min)	*tc*_90_ (min)
XE15	170	4.23	1.36	2.87	3.1	16.4
	180	5.34	1.59	3.75	1.4	2.7
	190	6.58	1.75	4.83	1.2	2.2
	200	8.34	1.83	6.51	0.9	1.8
XE15H3	170	4.46	1.56	2.9	3	13.3
	180	6.2	1.81	3.27	1.3	1.9
	190	8.29	1.89	6.41	1.2	1.7
	200	9.15	1.95	7.2	0.9	1.5
XE15H5	170	4.73	1.59	3.14	2.9	10.5
	180	6.57	1.91	4.66	1.2	1.7
	190	9.56	2.02	7.54	1	1.5
	200	10.93	2.12	8.8	0.9	1.4
XE15H7	170	5.49	1.62	3.88	2.8	9.3
	180	8.32	2.01	6.31	1.8	1.7
	190	10	2.09	7.9	0.9	1.3
	200	11.22	2.17	9.05	0.8	1.4

**Table 3:** Vulcanization kinetic parameters for various XNBR/epoxy/XHNT nanocomposites calculated through using n^th^ order model.

Designation	T (°C)	K (s^−1^)	n	lnA (s^−1^)	E_α_ (kJ/mol)
XE15	170	1.34	1.17	9.59	95.73
	180	1.42	1.18		
	190	1.54	1.27		
	200	1.68	1.35		
XE15H3	170	1.41	1.3	11.16	78.19
	180	1.48	1.38		
	190	1.57	1.45		
	200	1.73	1.53		
XE15H5	170	1.45	1.37	12.09	65.59
	180	1.51	1.42		
	190	1.63	1.46		
	200	1.76	1.58		
XE15H7	170	1.48	1.52	14.27	56.83
	180	1.54	1.66		
	190	1.65	1.69		
	200	1.78	1.85		

**Table 4:** Vulcanization kinetic parameters for various XNBR/epoxy/XHNT nanocomposites calculated through using the autocatalytic approach.

Designation	T (°C)	K (s^−1^)	m	n	lnA (s^−1^)	Eα (kJ/mol)
XE15	170	1.12	0.3	1.11	8.25	85.62
	180	1.13	0.36	1.13		
	190	1.15	0.44	1.21		
	200	1.23	0.47	1.23		
XE15H3	170	1.19	0.35	1.14	9.34	73.12
	180	1.25	0.4	1.18		
	190	1.29	0.49	1.25		
	200	1.33	0.52	1.27		
XE15H5	170	1.25	0.42	1.17	10.18	61.5
	180	1.3	0.48	1.22		
	190	1.34	0.53	1.26		
	200	1.38	0.58	1.28		
XE15H7	170	1.27	0.47	1.2	11.03	52.92
	180	1.32	0.56	1.24		
	190	1.37	0.6	1.27		
	200	1.43	0.66	1.32		

**Table 5:** Vulcanization kinetic parameters for various XNBR/epoxy/XHNT nanocomposites calculated through using the autocatalytic approach and isothermal DSC curves.

Designation	T (°C)	∆H (W/g)	∆H (W/g)	K (s^−1^)	m	n	lnA (s^−1^)	Eα (kJ/mol)
XE15	170	5.95	5.95	1.03	0.23	1.03	8.22	80.15
	180	6.35	6.35	1.05	0.27	1.08		
	190	8.47	8.47	1.07	0.3	1.13		
	200	10.36	10.36	1.11	0.32	1.18		
XE15H3	170	6.12	6.12	1.07	0.26	1.1	9.26	72.11
	180	7.54	7.54	1.1	0.3	1.13		
	190	8.86	8.86	1.12	0.33	1.17		
	200	10.59	10.59	1.13	0.36	1.21		
XE15H5	170	6.15	6.15	1.1	0.31	1.14	10.02	60.35
	180	7.66	7.66	1.12	0.36	1.16		
	190	9.03	9.03	1.16	0.38	1.21		
	200	10.77	10.77	1.18	0.42	1.26		
XE15H7	170	6.23	6.23	1.17	0.35	1.19	10.86	48.64
	180	7.82	7.82	1.2	0.41	1.22		
	190	9.18	9.18	1.23	0.45	1.28		
	200	10.85	10.85	1.25	0.48	1.3		

**Table 6:** Thermal decomposition characteristics of XNBR/Epoxy/XHNT nanocomposites calculated from TGA and DTG thermograms at heating temperature of 5 °C/min.

Designation	T5 (°C)	T10 (°C)	TP (°C)	WLP (%)	Residue (%)
XE15	355.83	382.26	456.54	53.04	24.47
XE15H3	356.00	382.87	461.26	49.19	30.30
XE15H5	357.79	383.69	462.25	46.67	30.87
XE15H7	360.0	383.8	468.3	44.20	32.40

**Table 7:** Degradation parameters obtained through using autocatalytic approach for various XNBR/epoxy/XHNT nanocomposites.

Designation		XE15	XE15H3	XE15H5	XE15H7
Friedman	E_α_ (kJ/mol.)	227.89	227.70	231.85	252.33
	Ln A (min^−1^)	27.52	33.85	34.96	35.42
	m	0.69	0.19	0.08	0.03
	n	2.28	2.17	1.65	1.12
KAS	E_α_ (kJ/mol.)	221.80	228.04	234.87	252.44
	Ln A (min^−1^)	29.65	30.95	34.27	35.20
	m	0.62	0.24	0.15	0.10
	n	1.76	1.74	1.63	1.54
OFW	E_α_ (kJ/mol.)	197.48	206.00	211.38	222.53
	Ln A (min^−1^)	29.87	31.15	34.28	35.17
	m	0.64	0.14	0.15	0.10
	n	1.89	1.77	1.75	1.33

## Data Availability

No new data were created or analyzed in this study. Data sharing is not applicable to this article.
